# Crack Classification of a Pressure Vessel Using Feature Selection and Deep Learning Methods

**DOI:** 10.3390/s18124379

**Published:** 2018-12-11

**Authors:** Manjurul Islam, Muhammad Sohaib, Jaeyoung Kim, Jong-Myon Kim

**Affiliations:** School of Electrical, Electronics and Computer Engineering, University of Ulsan, Ulsan 680-749, Korea; m.m.manjurul@gmail.com (M.I.); md.sohaibdurrani@gmail.com (M.S.); kjy7097@gmail.com (J.K.)

**Keywords:** fatigue crack detection, feature extraction, genetic algorithm, deep learning, pressure vessel, petrochemical industries, acoustic emission examination, nondestructive testing

## Abstract

Pressure vessels (PV) are designed to hold liquids, gases, or vapors at high pressures in various industries, but a ruptured pressure vessel can be incredibly dangerous if cracks are not detected in the early stage. This paper proposes a robust crack identification technique for pressure vessels using genetic algorithm (GA)-based feature selection and a deep neural network (DNN) in an acoustic emission (AE) examination. First, hybrid features are extracted from multiple AE sensors that represent diverse symptoms of pressure vessel faults. These features stem from various signal processing domains, such as the time domain, frequency domain, and time-frequency domain. Heterogenous features from various channels ensure a robust feature extraction process but are high-dimensional, so may contain irrelevant and redundant features. This can cause a degraded classification performance. Therefore, we use GA with a new objective function to select the most discriminant features that are highly effective for the DNN classifier when identifying crack types. The potency of the proposed method (GA + DNN) is demonstrated using AE data obtained from a self-designed pressure vessel. The experimental results indicate that the proposed method is highly effective at selecting discriminant features. These features are used as the input of the DNN classifier, achieving a 94.67% classification accuracy.

## 1. Introduction

Pressure vessels (PV) have widespread application in fields such as the petrochemical industry and nuclear energy [1,2,3,4]. Due to harsh operating conditions, pressure vessels can be dangerous and cause fatal accidents during their operation. Pressure vessel failures can happen due to corrosion, creep, fatigue cracking, and stress, of which fatigue cracking is the leading cause of the frequent failing of pressure vessels [5,6,7]. Therefore, it is evident that pressure vessel crack identification is an urgent task to prevent catastrophic accidents, as well as financial and environmental damage. The essence of a reliable crack identification scheme of a pressure vessel is composed of the following steps: fault feature calculation, discriminative fault feature analysis, and fault classification.

Pressure vessel cracks identification can be performed by collecting data (i.e., ultrasonic signals, eddy-current signals, thermal images, and acoustic emission signals), which has been an important aspect of studies conducted over the last couple of decades [1,8,9,10]. These fault identification studies prove that diagnosis of the pressure vessel can reduce maintenance expenses by enhancing the reliability of equipment. In the field of pressure vessel crack identification, ultrasonic signals and eddy currents have been widely exploited [4,10]. Alternatively, acoustic emission (AE) monitoring has gained significant attention recently in the field of pressure vessel monitoring since AE signals can capture intrinsic information from low-energy signals, even when the crack size is very small or structural deformation or cracks are not visible on the pressure vessel surface [11]. This makes AE techniques more suitable for early-stage crack detection [9,12]. Therefore, this study employs an AE-based crack classification approach for pressure in data-driven diagnostics.

Existing studies on pressure vessel monitoring systems are mostly based on crack detection in the form of spectrum visualization, but no classifier is used to identify crack types [13,14,15,16]. PV cracks are considered as highly nonlinear and non-stationary faults, which have many impacts on the signal. This makes it cumbersome to utilize conventional signal processing techniques, for instance, time-domain and frequency-domain analysis based on Fourier transform (FT), since they cannot correctly detect impulse phenomena in a non-stationary crack impact signal due to the inherent constraints of these approaches. To realize a highly reliable crack classification technique, it is essential to exploit discriminatory information from the measured data for complex industrial processes, such as those that use pressure vessels. We apply several signal processing techniques to detect intrinsic information about cracks from signals attained from the pressure vessel. These techniques are comprised of calculating different features in either the time domain, frequency domain, or time-frequency domain. A feature extraction process based on a single method may overlook the discriminative properties of crack conditions [15,17,18]. One of the major contributions of this study is constructing a heterogeneous feature pool consisting of three simultaneous feature extraction models: the time domain, frequency domain, and time-frequency domain. 

In addition to time and frequency domain features, time-frequency analysis (TFA)-based features are also appropriate for pressure crack classification since TFA simultaneously analyzes a measured signal concerning the crack in both the time and frequency domains where impulse information is detectable [18,19]. One of the most widely used TFA methods for pressure vessel signal analysis is called the wavelet transform. The obvious advantage of wavelet-based signal processing techniques is that they have a good time-frequency localization, which allows for the detection of transients that appear in the signals. Thus, we employ the wavelet pack transform (WPT), which is highly effective at decomposing the signal into mid- and high-band frequency nodes (e.g., sub-bands) so that crack information can be observable. For this reason, we extract energy information in the mid- and high-band frequency nodes of WPT.

Thus, the fusion of the proposed heterogeneous feature models from multiple sensors is significantly a high-dimensional feature vector involving as much information as possible about the process conditions that ensure the availability of all necessary information concerning accurate identification [20]. However, there is still an issue of selecting the most discriminant features that contain essential information about the mechanical crack being investigated. In practice, however, high-dimensional feature vectors involving a large number of features are either irrelevant or redundant to the aforementioned predictive models (supervised and unsupervised classifiers). These irrelevant or redundant features can be a reason for the degraded classification performance of a modern crack identification technique. To alleviate this issue, discriminatory feature selection is an indispensable part of the pressure vessel crack identification method, and the main goal of this feature selection is to attain a refined subset of a discriminatory feature from the original high-dimensional feature vectors. To achieve a highly efficient identification performance, quite a few PV crack identification methods adopt feature selection. A global similarity scheme has been presented to select informative features that could be helpful for identifying cracks for a spherical tank [21]. Researchers also validated the efficacy of the feature selection technique that combines kernel feature selection and principal component analysis (PCA) for pressure vessel applications [15,22,23]. 

Consequently, this paper proposes an intelligent pressure vessel crack classification method that combines a genetic algorithm (GA) with heterogeneous feature models from several channels to select the most discriminative feature, and this selected subset is then used with the deep neural network (DNN) for classifying cracks. To allow GA to autonomously select a set of discriminant features, a new fitness function that utilizes the ratio of the within-class distance (WCD) of the pressure vessel crack classes and between-class distance (BCD) is introduced in this study. The quality of the GA results is strongly dependent on the designed fitness function. Thus, the proposed fitness function, $\frac{BCD}{WCD}$, calculates the distances between-classes and within-class to ensure maximum separability among crack classes. 

Although GA helps to select a discriminant features subset, real-world applications such as pressure vessels may have various complexities in their feature distribution. Therefore, we employ DNN for improving the classification performance. In contrast with traditional classifier techniques such as the support vector machine [18] and k-nearest neighborhood [23], DNN can minimize the dimensionality of data representation and recognize targets [24] effectively. Consequently, DNN can be trained to learn useful feature information, even if the proposed GA generates a features subset with a complex distribution. Therefore, the effectiveness of the proposed method (GA + DNN) is validated using AE data collected from a pressure vessel.

The upcoming sections of the paper are structured as follows. Section 2 contains details of the materials and methods, including the experimental setup, feature extraction, feature selection, and crack classification techniques. Section 3 presents the acquired results, justifies the efficacy of the proposed approach with the required graph, and matches its efficiency to the previously accepted methods. Finally, Section 4 concludes the entire study by summarizing the paper.

## 2. Material and Methods

**Figure 1:**
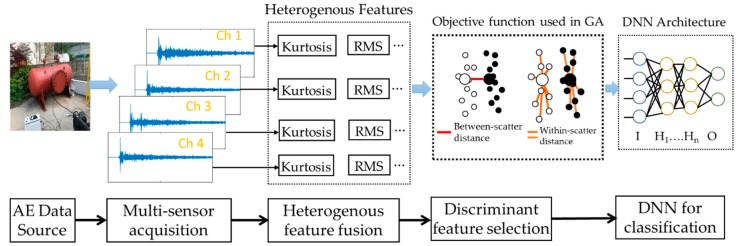
Flowchart of the proposed method for pressure vessel crack classification. In the figure, Ch: Channel, I: Input layer, H: Hidden layer, and O: Output layer.

This study aims to classify pressure vessel cracks based on heterogeneous feature models from multiple sensors and a deep neural network (DNN). As declared in the previous section, the method we are presenting can be broken down into four discrete steps: AE data acquisition, hybrid feature extraction, discriminant feature selection using GA with a new objective function, and a deep neural network for classifying cracks. The block diagram of the proposed methodology is given in Figure 1.

### 2.1. Pressure Vessel Experiment System and AE Data Acquisition

**Figure 2:**
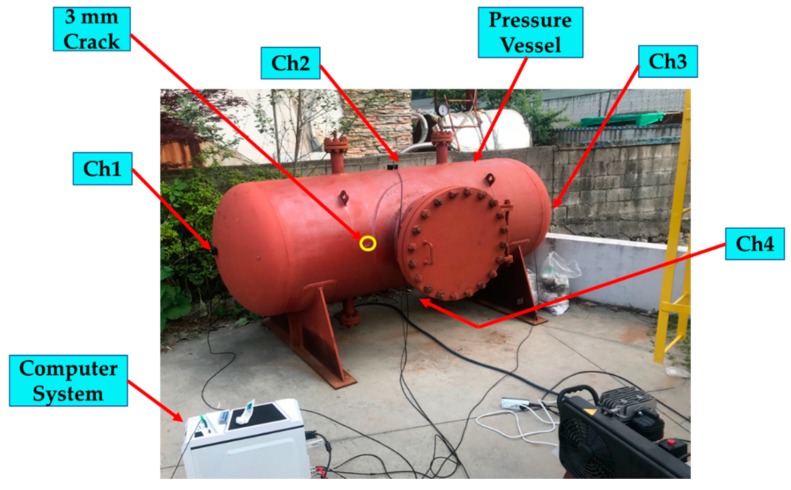
Pressure vessel test rig and its component locations that were used for data acquisition.

**Figure 3:**
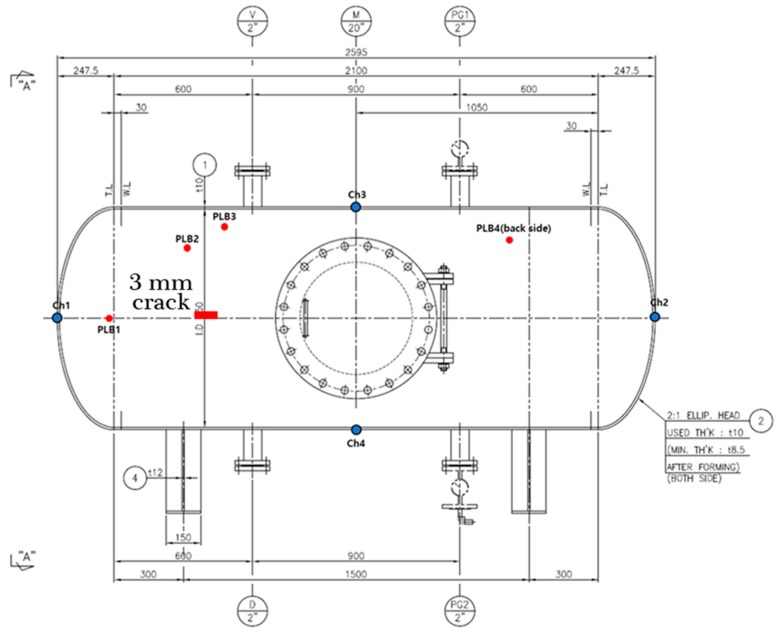
Geometric diagram of the pressure vessel with the specific positions of the channels and crack location.

**Figure 4:**
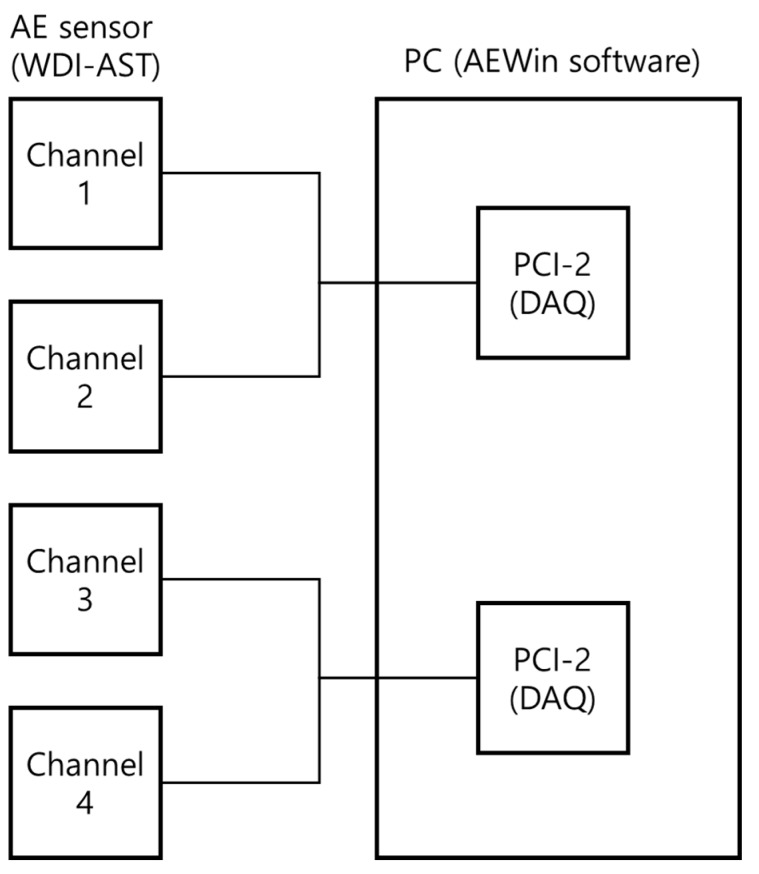
The channels (sensors) arrangements for the data acquisition system.

To verify the effectiveness of the proposed acoustic emission (AE)-based method, we conducted experiments using a data acquisition system specified by engineering norm ASME BPVC.V-2015 (American Society of Mechanical Engineers (ASME) Boiler & Pressure Vessel Code (BPVC)), including a recent study on pressure vessel fault diagnosis [25]. The pressure vessel test rig used included a pressure vessel, AE sensors, channel information, the PCI system, and a computer system, as depicted in Figure 2. To explain details about the experiment and data acquisition system, we created a pressure vessel dataset in two conditions: normal and artificially induced cracks. To collect fault condition data, a 3 mm crack was manually induced on the surface of the pressure vessel, as can be seen in Figure 3. Four AE sensors were attached to the surface of the pressure vessel at different locations, based on ASME BPVC.V-2015 experiment design guidelines. A pencil lead break (PLB) test was performed to generate a guided wave through the pressure vessel surface [26]. Velocity acoustic emission signals were recorded using AE sensors. On the other hand, we collected normal condition data from a healthy pressure vessel (e.g., no crack in the surface). The arrangement of channels (sensors) during the experiment is shown in Figure 4. The AE signals were recorded at a 1 MHz sampling frequency. Multiple samples were recorded, each for 0.1 s. The dataset is described in Table 1.

### 2.2. Heterogeneous Feature Extraction

As explained in Section 1, most of the existing studies only focus on the crack detection problem, and few studies have employed a traditional classifier (e.g., SVM) with a signal feature model to identify fault types [18]. We, therefore, extracted heterogenous features from various signal processing domains, namely the time domain, frequency domain, and WPT. The main idea of such diversity in the feature extraction process is so that no information about the crack is missed. These features are regarded as discriminative since there is a significant change in the magnitude of the signal when impulses occur due to a crack in the pressure vessel. Therefore, the changes in signal behavior due to a crack can be well-characterized by extracting time-domain statistical feature parameters, such as the root mean square (RMS) (F1), kurtosis (F2), skewness (F3), and impulse indicator (F4). All the time-domain features used provide statistical properties about the nature of data and were found to be reasonably good features for PV cracks because they were sensitive to impulse faults [17,27]. 

Furthermore, the frequency-domain feature can also reveal some important information that cannot be observed in the time domain [17]. Several studies [18,28] have revealed that the frequency spectrum of the original signals obtained by fast Fourier transform (FFT) provides additional information about the crack, which is helpful for classifying pressure vessel cracks. Thus, the frequency-domain features extracted in this study are as follows: frequency root mean square (F5), frequency standard deviation (F6), and mean frequency (F7). Seven extracted features in the time domain and frequency domain are given in Table 2.

**Figure 5:**
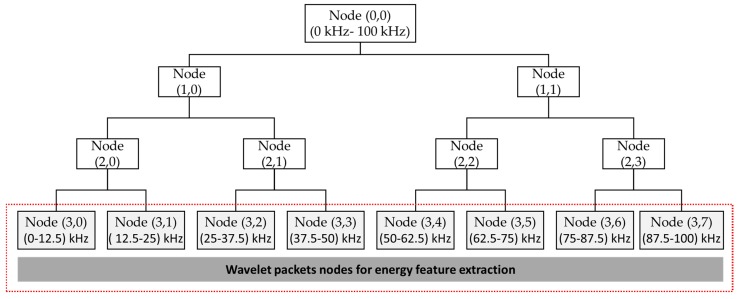
The decomposition method of WPT up to three levels for extracting energy features.

In addition to time-domain and frequency-domain features, we applied the wavelet pack transform (WPT) pressure vessel signal for obtaining the time-frequency domain features. WPT is highly effective at decomposing the signal into mid- and high-band frequency nodes (e.g., sub-bands) in which crack information can be observable. For this reason, WPT is applied with a 0.1 s AE signal to extract energy information in the mid- and high-band frequency sub-bands. According to Kang et al. [19], the relative energy in the WPT (REWPE) sub-bands is highly effective for revealing the disordered behavior of the signal due to a crack in the pressure vessel steel. To compute these energy features, we applied a three-level WPT, and we had eight sub-bands, as shown in Figure 5. Furthermore, the Daubechies 20 (or dB 20) mother wavelet function was used in this study during the WPT decomposing operation. Therefore, REWPE can be designed for each node as follows:

(1) $$REWPE\left(k\right)=\frac{\sum_{i=1}^{L}w_{k,j}^{2}}{\sum_{n=1}^{N_{tnodes}}\sum_{i=1}^{L}w_{k,j}^{2}}$$

where $N_{tnodes}$ is the total number of WPT nodes (e.g., $N_{tnodes}$ = 8 in this study), *L* is the number of wavelet coefficients for each node, and $w_{k,j}$ is the *j*-th wavelet coefficient of the *k*-th node.

We calculated the REWPE value for each of the eight WPT sub-bands in the 3rd level, which are denoted as features F7-F15. Therefore, we obtained 15 features, including four time-domain, three frequency-domain, and eight REWPE values for each channel signal. As our main target is to conduct multi-sensor feature fusion to ensure the availability of all information about a crack, we obtained a total of sixty features for four channels that were used in the GA for selecting the most discriminant feature. 

### 2.3. Discriminant Feature Selection Using GA

The fusing of heterogeneous features from four channels can be redundant and irrelevant due to large dimensionality [23,29], so selection of the most meaningful features that contain discriminant information about pressure vessel cracks is inevitable. The optimal subset can be determined in three ways, namely through complete, sequential, and heuristic searches [29]. Although a complete search provides an optimal subset since it applies a brute-force search, the computational complexity of this approach is high. In contrast, a sequential process is comparatively fast, but it does not guarantee the best results. Heuristic approaches, including a genetic algorithm (GA), offer a good tradeoff between the computational complex and the quality of the selected feature subset [23]. Therefore, this study deploys GA for selecting the discriminant feature subset that is highly effective for representing pressure vessel cracks.

**Figure 6:**
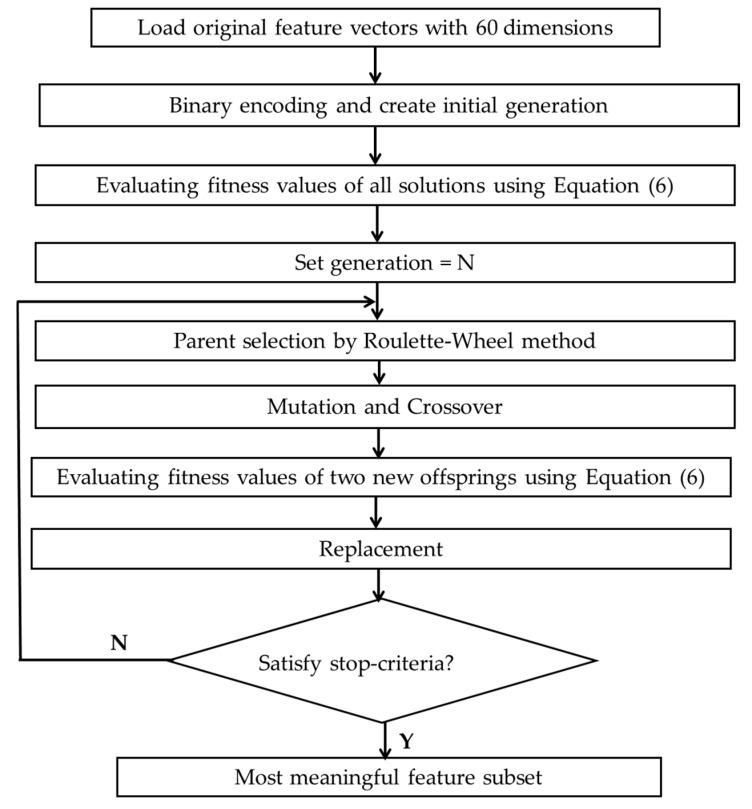
The flow diagram of the genetic algorithm (GA) procedure for finding an optimal IMF set.

The GA is applied to generate a high-quality solution in optimization problems based on natural selection, which is comprised of specific discrete steps, such as problem representation (encoding), parent selection, crossover and mutation, and replacement. The best solution is produced in the form of the chromosome, which is a combination of genes. This paper uses a generational GA: in every generation, n offspring are created, and the low-quality chromosomes in the population are replaced with those of the newly generated offspring. The flow diagram of GA is given in Figure 6.

In the proposed GA-based discriminant feature selection, we use binary encoding, roulette-wheel parent selection, one-point mutation, and uniform crossover. Specifically, we created 300 initial populations using the binary encoding technique, and the length of each chromosome is equal to the number of features (e.g., 60 in this study). Each chromosome denotes a set of zeros and ones, where ones are randomly assigned to feature components and zeros are assigned to not selected features. For example, the chromosome view of 60 features is $0^{1}100000001\cdots \cdots 000^{60}$, which means the 2nd and 10th features are selected in the current solution. One-point mutation and uniform crossover are utilized to reduce the chance of separating the closely located genes in the selected parent chromosome during the recombination process.

In this paper, we use a total of 1000 generations, and for each generation, 50 offspring are created, and 50 chromosomes with the worst fitness values in the population are replaced with those that are newly generated. These parameters are defined experimentally based on a high system performance. However, the quality of the GA results is strongly dependent on the designed fitness function. To define a fitness function for GA, Kang et al. analyze the crack classes, including the average distance-based feature evaluation metric, which does not consider the complexity of class and overlooks the overlap in between class distances significantly [29]. In this study, we define an improved evaluation metric as the ratio of the within-class compactness and between-classes separation, as determined by the average Euclidean distance-based approach, is not always sufficient to fully describe the distribution of samples of all classes [23]. In this study, we define an improved evaluation metric as the ratio of the within-class distance (WCD) of crack classes to the between-class distance (BCD) that carefully analyzes the distances between-class and within-class to ensure maximum separability among crack classes. In the case of WCD and BCD calculations, we use the center median instead of the average-distance for each class (i.e., crack category in this study) for perfect work on both Gaussian and non-Gaussian feature distribution. The fitness function calculation for GA is depicted in Figure 6. Thus, WCD can be calculated as follows: 

1. Calculate distances between all samples within the class as follows:

Let $d=d_{1},d_{2},d_{3},\ldots ,\;d_{n}$ be a set of data points in a class, where *n* is the total number of data points in the class. In addition, each data point in *d* corresponds to a vector involving a number of fault features, such as F. Find the centroid of each class, such as $C_{i}$. Now, calculate the Euclidian distance all datapoints associated with centroid, C, as follows:

(2) $$L_{i}=\sqrt{\sum_{j=1}^{F}\left(C_{i}-d_{i_{j}}\right)},\;i=1,2,\ldots ,n$$

2. Find the maximum distance associated with the centroid, $D_{c}$, as follows:

(3) $$D_{c}=\underset{i}{\arg\max}\left\{L_{i}\right\}$$

3. Finally:

(4) $$WCD=\frac{1}{N}\sum D_{c}$$

where *N* is the number of classes (e.g., two in this study).

*BCD* is a distance measure that first calculates the center median of all classes and then takes the average distance of one to the rest of the class. *BCD* can be calculated as follows:

(5) $$BCD=\frac{1}{N}\sum_{i=1,i\neq j}^{N}C_{i,j}$$

where $C_{i,j}$ measures the Euclidean distance from class i to class j where i≠j.

Now that the *WCD* and *BCD*-based feature evaluation metrics are ready, we define a function in a form, which utilizes *WCD* and *BCD*, to maximize the fitness function (as the ratio of the maximum value of *BCD* and the minimum amount of *WCD*) as follows:

(6) $$fitness=\frac{BCD}{WCD}$$

**Figure 7:**
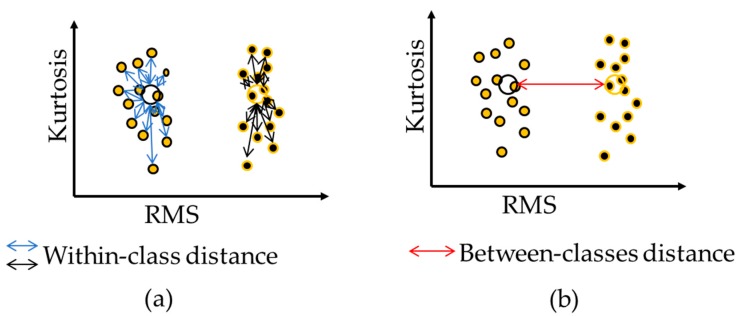
The fitness function calculation for a genetic algorithm (GA): (**a**) shows the within-class distance measure and (**b**) shows the distance measure of the between classes. In the figure, big circles indicate the center median of each class.

The defined fitness in Equation (6) is highly effective, and simultaneously tries to maximize the distances between classes and minimize the distances within classes, as shown in Figure 7, which ultimately results in a discriminant features subset with maximum separable distributions while this fitness is used with a state-of-the-art optimization algorithm such as (GA).

### 2.4. DNN for Classifying Cracks

Though GA provides a simple distribution of crack classes, we still considered a robust classifier technique for classifying cracks since, for practical applications, pressure vessel crack classes may have complex distributions. In this study, we applied a deep neural network (DNN) after GA selection for classifying cracks. There are various types of neural network architectures, and one of the most common is the Multi-Layer Perceptron (MLP) with multiple hidden layers [24,30].

**Figure 8:**
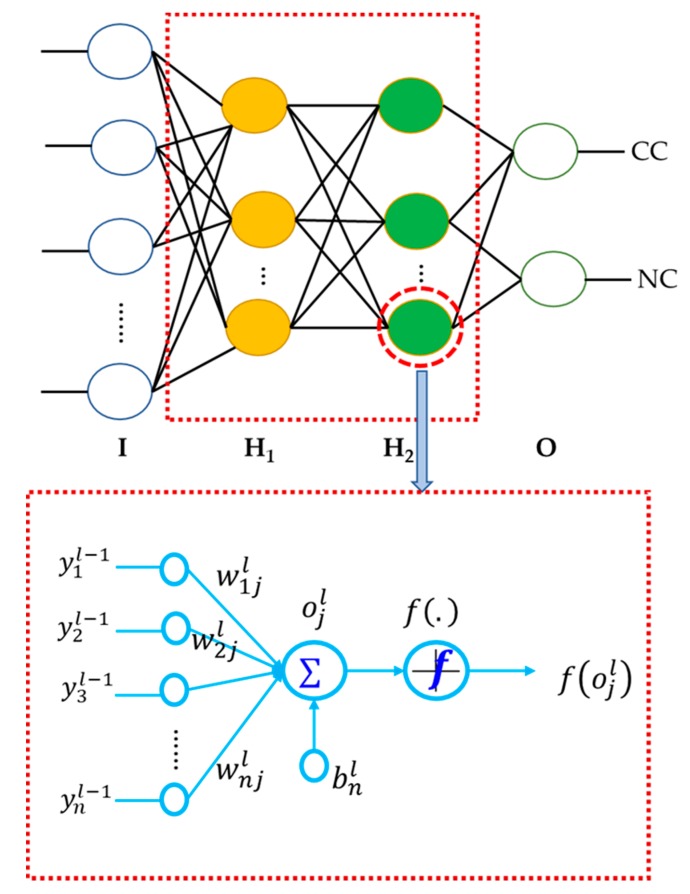
Proposed DNN for identifying pressure vessel cracks. In the figure, I: Input layer, H: Hidden layer, O: Output layer, CC: Crack Condition, NC: Normal Condition, and F: Feature value for the input.

DNN is a stacked layer model in which the layers are connected subsequently, and there are no connections of nodes within the same layer [24]. DNN includes an input layer, an output layer, and a few hidden layers placed between them in the model, as can be seen in Figure 8. The number of nodes of an input layer is set corresponding to the dimensionality of the input data. Likewise, the number of nodes of an output layer is defined corresponding to the dimensionality of the target data. The number of nodes of every hidden neural layer is set by the network function, for which there are no required strict regulations. Each node in the next layer is directly linked to all nodes in the previous layer. Nodes of the first layer receive the input data and transmit them to other layers, while nodes of the last layer output the targets. The nonlinear relationship between the DNN layers is indicated by the following equations:

(7) $$o_{j}^{l}=\sum_{i}y_{i}^{l-1}w_{ij}^{l}+b_{j}^{l},$$

(8) $$h_{W,b}\left(y\right)=y_{j}^{l}=f\left(o_{j}^{l}\right)=f\left(\sum_{i}y_{i}^{l-1}w_{ij}^{l}+b_{j}^{l}\right),$$

where $y_{j}^{l}$ is the activation value of neuron *j* in layer *l*; $o_{j}^{l}$ is a linear activation combination of neurons in the previous layer; $b_{j}^{l}$ is the bias value of neuron *j* in layer *l*; $w_{ij}^{l}$ is the weight parameter between nodes *i* in layer *l* − 1 and *j* in layer *l*; and f(·) is the activation function, which is usually chosen to be logit and mostly used in DNN.

As the backpropagation (BP) algorithm is applied to train DNN, the gradients of the loss function for all trainable weights in all layers are calculated during the backward operation of BP [30]. However, it is essential to define an appropriate objective function. Thus, a squared-error loss function is applied to address the objective function. Equation (9) defines the loss function after training a single sample, such as *i*. The overall loss function can be calculated by summing the loss functions of each sample, as defined below:

(9) $$cost(w)=\frac{1}{2}{\sum_{i=1}^{m}\left({o_{i}}^{l}-t_{i}^{l}\right)}^{2},$$

where $t_{i}^{l}$ defines the target output value of the $i^{th}$ pattern.

Suppose a point *w* to find the next weight point (*w* + 1) to find a minimizer. It starts from *w* and moves by $\alpha \frac{\partial }{\partial w}cost(w)$, as in Equation (7), where α is a positive scalar step size.

(10) $$w:=w-\alpha \frac{\partial }{\partial w}cost(w)$$

The weight update process in Equation (10) is called a stochastic gradient descent (SGD) algorithm. Once the training operation of DNN is completed, the optimized parameters are used for verifying the proposed pressure crack classification scheme. 

## 3. Results and Discussion

The effects of two main components of the proposed pressure vessel crack identification scheme (GA + DNN)—GA-based discriminant feature selection and the DNN classifier for improved diagnostic performance— are analyzed and discussed in this section.

**Figure 9:**
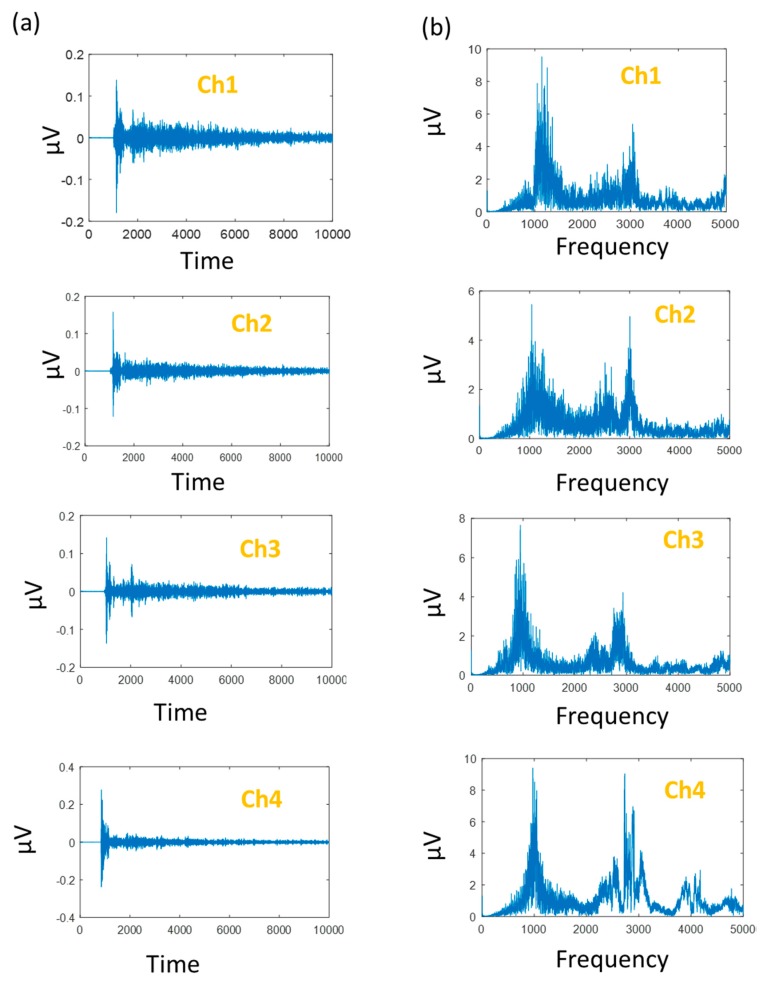
(**a**) Time-domain pressure vessel AE signals and (**b**) corresponding frequency-domain signals of the signals in (a).

The proposed method is examined using pressure vessel AE data gathered from a self-designed test rig (see Table 1). In this dataset, we have 90 samples of each crack category (i.e., two in this study) for each channel (e.g., four in this study). The effectiveness of the data acquisition method can be seen in Figure 9, showing a time-domain signal of each channel and their corresponding frequency-domain signal.

### 3.1. Performance Evaluation of GA-Based Discriminant Feature Selection

**Figure 10:**
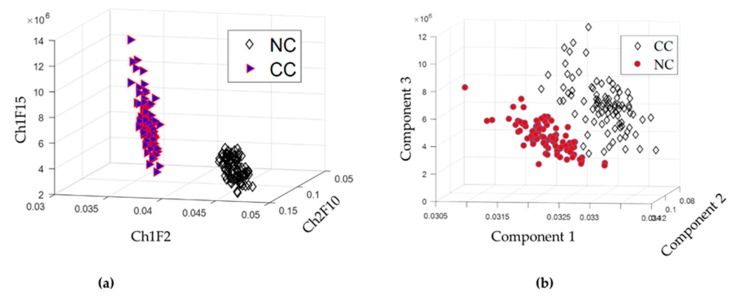
3D visualization of the selected feature for (**a**) the proposed GA-based feature selection and (**b**) the first three components of PCA [23].

One of the main contributions of the proposed method is the selection of essential features using an appropriate fitness function for GA. To validate the performance of the proposed GA-based feature selection, we compared the proposed GA with that of conventional principal component analysis (PCA) [23]. Table 3 summarizes the result of the selected valuable feature of GA with a proposed fitness function. According to the results in Table 3, the proposed GA-based is highly able to refine a high-dimensional feature vector into a smaller number of features from the original 60 feature vectors. The effectiveness of the selected features can be verified in Figure 10 in 3D visualization for the proposed GA and PCA. For component analysis, this study explores the effect of the principal component in terms of classification accuracy since the first n component generates the highest performance that is used in practice for PV crack classification.

### 3.2. Performance Evaluation of DNN for Improved Classification Accuracy

In this study, although GA helps us to obtain a discriminant features subset, real-world applications such as pressure vessels may have various complexities in their feature distribution. Therefore, we employed a DNN classifier for improving the classification performance that can effectively work on minimizing the dimensionality of data representation and recognize targets correctly.

To authenticate the performance of the proposed method, it is essential to divide the dataset into appropriate training and testing for DNN performance measurement. We randomly divided our data into 33.33% for training, 16.67% for validation, and the remaining 49.995% for testing. In terms of the number of samples, we had 30 samples for training, 15 samples for validation, and the remaining 45 samples for testing. The test dataset was kept higher than that of training to generalize the classification performance. Thus, to evaluate the usefulness of the proposed method in classifying pressure vessel cracks and confirm the advantages of the GA-based feature selection process, we compared our methodology with the state-of-the-art approaches that utilize PCA and hybrid fault features for classifying faults using the k-NN classifier. This method is referred to as PCA + k − NN [23]. Another comparison method extracts the proposed heterogeneous features directly from the raw AE signal (referred to as All-Features). The classification performance is calculated through the average classification accuracy (ACA) as follows:

(11) $$ACA=\frac{TP+TN}{TS}$$

where *TP* (true positive) defines the number that was correctly classified as the predicted class, *TN* (true negative) defines the number of correct negative predictions, and *TS* defines the total number of samples that were used in this experiment. 

**Figure 11:**
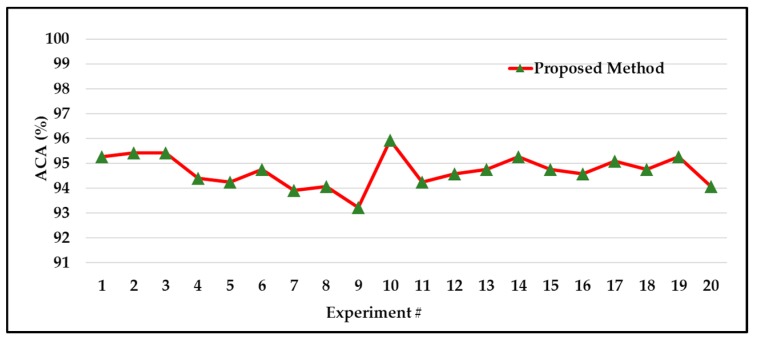
Classification accuracies of the proposed method achieved over 20 experiments.

Table 4 presents the experimental results for three models. According to the results in Table 3, the proposed classification method (GA + DNN) outperforms the referenced methods regarding the average ACA, with a value of 94.69% achieved over 20 experiments. According to the results shown in Table 4, it is evident that the proposed (GA + DNN) method outperforms the two referenced methods, yielding 12.28% and 3.32% performance improvements for All-Features and PCA + k − NN, respectively. Further, we provide the results of 20 experiments for the proposed method in Figure 11.

**Figure 12:**
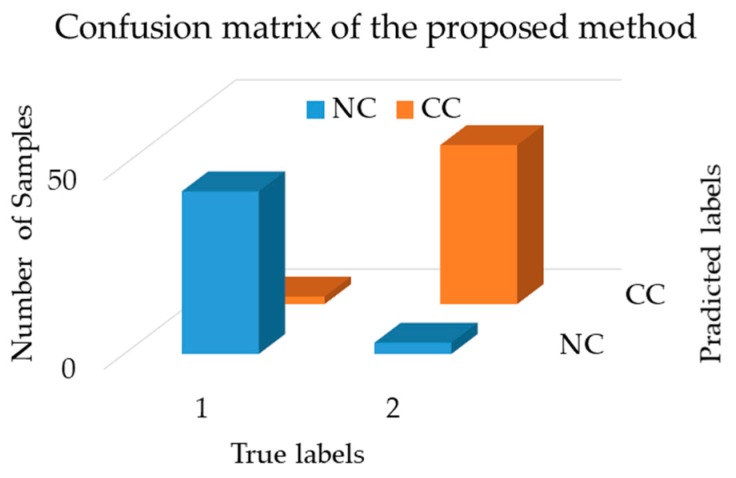
Confusion matrix showing the classification of the proposed method.

**Figure 13:**
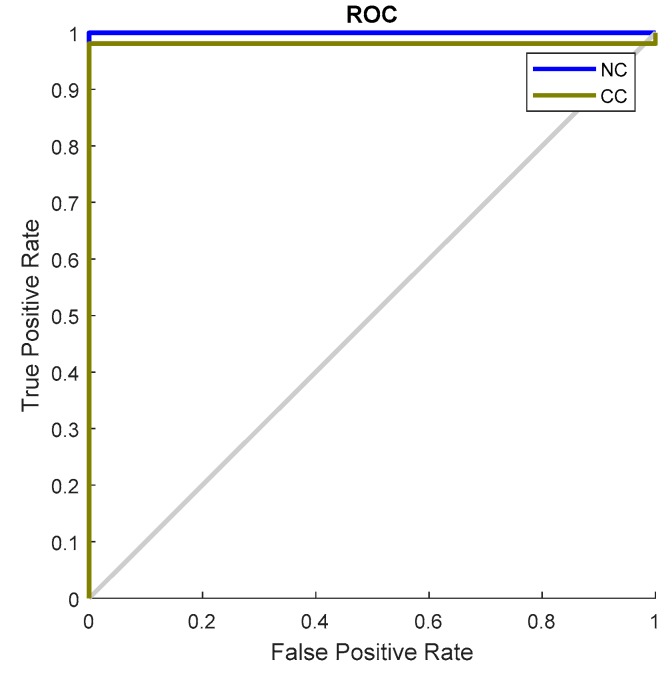
AUC-ROC of the proposed DNN classifier.

Additionally, we provide the result of the confusion matrix for the proposed framework and reference methods. The confusion matrix is a reliable way to judge any supervised learning algorithm (e.g., DNN) because it provides a visual image where the actual labels and the predicted deviation can be audited. Figure 12 shows the confusion matrix of our proposed method, which indicates that the technique is capable of correctly identifying cracks with a negligible misclassification rate. Figure 13 presents the receiver operating characteristic (ROC) curve to illustrate the tradeoff between the sensitivity and specificity of our model. As we can see, the curves of all four classes follow the left and top border of the ROC space, meaning the classifier result is highly accurate.

**Figure 14:**
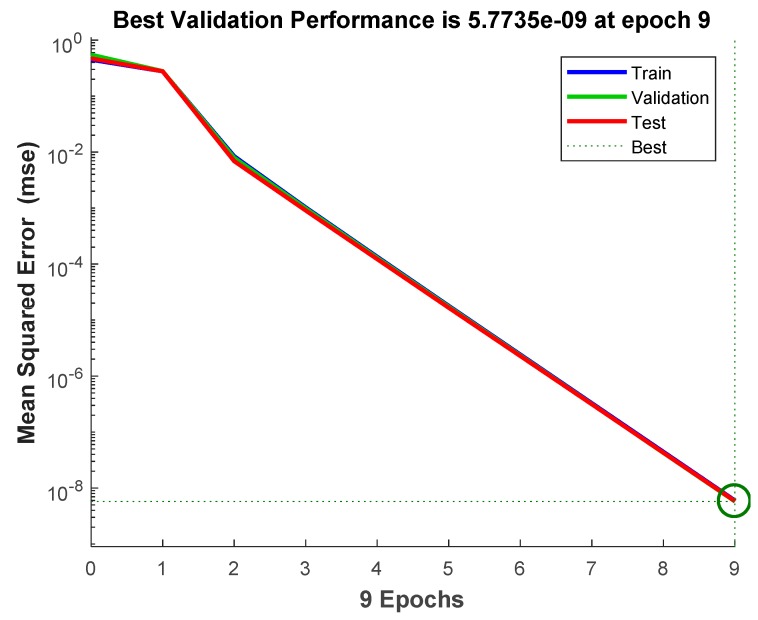
Training performance of the proposed DNN classifier.

For a detailed analysis of the DNN network for an improved classification performance, one obvious observation is that the proposed DNN is highly effective at reaching a near optimum value of SGD optimization from the epochs learning, which proves that the DNN-based approach can yield the desired accuracy faster, as can be seen in Figure 14.

Overall, the proposed methodology is highly effective because of its two main conceptions: GA-based feature selection with an appropriate fitness function and the further application selected features subset in the DNN classifier with proper parameter setting.

## 4. Conclusions

This study developed a new method of crack identification of a pressure vessel, which is composed of crack feature calculation, GA-based discriminative feature selection, and a deep neural network (DNN) for classifying cracks in an acoustic emission (AE) examination. The proposed method first extracts heterogeneous features from multiple sensors that represent diverse symptoms of pressure vessels faults. However, hybrid features from different channels are a significantly large dimension that carries redundant and irrelevant features. This study selects the most discriminative features using GA with a new objective function—the ratio of the within-class distance (WCD) and between-class distance (BCD)—to improve the classification performance. Finally, DNN was used with selected features for classifying pressure crack types. The potency of the proposed method (GA + DNN) was validated using the AE data obtained from a self-designed pressure vessel test rig. The experimental results demonstrated that the proposed method was highly effective at selecting discriminant features that contribute to achieving a 94.67% identification performance, while the selected features are used with a DNN classifier.

## Figures and Tables

**Table 1:** Dataset description.

Signal Type	Crack Size (mm)	Number of Samples	Number of Channels
Normal	0	90	4
Crack	3	90	

**Table 2:** Definition of the time-domain and frequency-domain statistical parameters (i.e., features) of this study.

Features	Equations	Features	Equations	Features	Equations	Features	Equations
Time-domain statistical features							
F1	$\sqrt{\frac{1}{N}\sum_{i=1}^{N}x^{2}(i)}$	F2	$\frac{1}{N}\;\sum_{i=1}^{N}\;{\left(\frac{x(i)-\bar{x}}{\sigma }\right)}^{4}$	F3	$\frac{1}{N}\;\sum_{i=1}^{N}\;{\left(\frac{x(i)-\bar{x}}{\sigma }\right)}^{3}$	F4	max|x(n)|1N∑n=1N|x(n)|
Frequency-domain statistical features							
F5	$\sqrt{\frac{1}{N}\sum_{i=1}^{N}f^{2}(i)}$	F6	$\sqrt{\frac{1}{N}\sum_{i=1}^{N}{\left(f(i)-f_{5}\right)}^{2}}$	F7	$\frac{1}{N}\sum_{i=1}^{N}f(i)$		

where x is an original AE signal in a time domain and f is the frequency domain signal of x.

**Table 3:** Summary of the discriminant feature subset attained by the proposed GA. In this Table, for example, Ch1F1 means feature 1 (i.e., RMS) of Channel 1.

Methodology	The Most Discriminant Feature Subset
GA with new fitness function	{Ch1F1, Ch2F10, Ch1F15, Ch3F1, Ch2F8, Ch4F1, Ch1F8}

**Table 4:** Average classification of the three different models.

Methodologies	ACA (%)
All-Features	82.83
PCA + k − NN [23]	91.79
GA + DNN (Proposed)	94.67
